# Lifespan trajectories of the brain’s functional complexity characterized by multiscale sample entropy

**DOI:** 10.1016/j.neuroimage.2026.122085

**Published:** 2026-07-01

**Authors:** Dilmini Wijesinghe, Kirsten Lynch, Leon Aksman, Dean C. Delis, Danny JJ Wang, Kay Jann

**Affiliations:** a USC Stevens Neuroimaging and Informatics Institute, Keck School of Medicine at USC, Los Angeles, CA, USA; b Department of Psychiatry, University of California San Diego, San Diego, CA, USA

**Keywords:** Resting-state fMRI, Complexity, Multiscale sample entropy, Lifespan trajectories, Cognition

## Abstract

Resting state functional magnetic resonance imaging (rs-fMRI) is a widely used imaging modality that can capture spontaneous neural activity of the brain. The human brain is a complex system, and emerging evidence suggests that the complexity of neural activity may serve as an index of its information processing capacity. In this study, we used multiscale sample entropy (MSE) to analyze the complexity of rs-fMRI time series from 504 healthy subjects aged 6 to 85 years. We constructed global and regional trajectories of the brain’s functional complexity across the lifespan and examined its correlation with executive function. We observed a nonlinear trajectory of fMRI-complexity, with a peak occurring at 23 years of age (95% CI: 21.27,26.38 years). Males reached the peak complexity at 26 years (95% CI: 19.95, 33.14 years), whereas females peaked at 23 years (95% CI 20.16, 29.18 years). Significant correlations were found between complexity and Number-Letter Switching of the Trail Making Test in the parietal and medial temporal lobes, while Inhibition and Inhibition/Switching of the Color Word Interference Test showed significant negative correlations with rs-fMRI complexity in the lateral and medial frontal cortex. These results provide insight into the behavior of fMRI-complexity across lifespan stages and highlight its association with executive function. As a non-invasive biomarker, fMRI-complexity may offer a novel approach to understanding the brain’s information processing capacity and its deficits in illness.

## 1. Introduction

Brain maturation is a complex and protracted process involving both progressive and regressive events, playing a critical role in cognitive development. During this process, individuals undergo significant physiological, psychological, and biological changes. Understanding normal developmental and aging processes across the lifespan is crucial for establishing a foundation for the detection, prevention, and treatment of brain disorders related to aging, as well as neurodevelopmental disorders that may have lifelong impacts (Cainelli and Bisiacchi, 2022; Tenchov et al., 2024). Establishing typical developmental trajectories is essential for identifying deviations indicative of psychiatric and neurological disorders. Such deviations may be critical for predicting age-related cognitive decline and for identifying biomarkers that enable early detection of neurodevelopmental disorders and neurodegeneration (Guan, Jiang, et al., 2023).

The human brain consists of neurons, synaptic connections, and networks of neuronal populations that exhibit hierarchical organization and give rise to nonlinear, dynamic, and complex behaviors. Over the years, efforts to understand brain development and aging have employed a variety of modalities. Structural magnetic resonance imaging (MRI) methods are widely used to assess metrics such as cortical thickness and volume, while diffusion-weighted imaging is employed to study the maturation of white matter fiber tracts (Frangou et al., 2022; Hedman et al., 2012). Resting-state fMRI (rs-fMRI) has been used to investigate intrinsically fluctuating signals and interactions between different brain regions (S. M. Smith et al., 2013). Functional connectivity analyses of fMRI data have revealed that the brain relies on stable networks to process information from the external environment (Biswal et al., 2010).

In information theory, information processing capacity (the uncertainty of information) can be quantified using statistics-based entropy measures. These nonlinear and dynamic methods are well-suited for analyzing the complexity of fMRI time series data. Recent advances in complexity metrics have enabled their application to fMRI signals as voxel-wise indicators of regional information processing capacity. Thus, applied to resting-state fMRI, voxel-wise entropy estimation allows for quantification of spatially resolved brain signal complexity ranging from the voxel level to regions and networks to global brain complexity. Brain complexity is often interpreted as reflecting fluctuations among brain networks, since entropy represents the number of possible configurations a system can assume (Saxe et al., 2018), therefore complementing conventional functional connectivity approaches. Accordingly, we hypothesize that regions with higher complexity possess greater adaptability, enabling more flexible information processing across networks and supporting cognitive function.

Over the past decade, several fMRI studies have examined how brain complexity changes with age using different metrics such as fractal dimensions or entropy-based methods (Dong et al., 2018; Niu et al., 2022; Sokunbi et al., 2015). Overall, these studies report a general decline in complexity with age. Beyond typical developmental and aging trajectories, evidence from neurodevelopmental and aging-related disorders suggests that brain entropy can detect deviations from normative patterns (Jann et al., 2024; R. X. Smith et al., 2018; Sokunbi et al., 2013; Zhang et al., 2024). Furthermore, according to our hypothesis, brain entropy reflects transitions and communication both within and between networks. Higher-order cognitive abilities, such as adaptive responses, reasoning, learning, problem solving, and planning, require accessing a large repertoire of brain states to integrate information effectively (Saxe et al., 2018). This raises important questions about whether complexity derived from neuroimaging reflects general intelligence or cognitive performance in specific domains. Addressing these questions requires additional studies to clarify the physiological significance of complexity (D. J. J. Wang et al., 2018), its normal developmental trajectories, and its relationship to cognitive function.

In this study, we investigate voxel-wise changes in multiscale sample entropy (MSE) across developmental stages and explore sex differences. MSE is an extension of Sample Entropy (SampEn) that quantifies the uncertainty or irregularity in a dynamical process (Costa et al., 2005; Omidvarnia et al., 2021; Richman and Moorman, 2000). MSE is particularly suitable for fMRI time series, as it can differentiate random noise from structured, self-similar processes (Richman and Moorman, 2000; R. X. Smith et al., 2014) and has been shown to produce reproducible measures of temporal complexity (Omidvarnia et al., 2021). However, MSE has not yet been applied systematically to study development and healthy aging across the entire lifespan using rs-fMRI. Additionally, to test our hypothesis that higher voxel-wise brain complexity reflects greater flexibility of a region in processing information, we examined the relationship between voxel-wise fMRI complexity and age-dependent changes in executive cognitive function, a higher-order cognitive domain primarily associated with frontal lobe activity (Keifer and Tranel, 2013).

## 2. Materials and methods

### 2.1. Ethics statement

Data used in this study were obtained from publicly available neuroimaging repositories and were fully de-identified prior to analysis. The investigators contributing to the original datasets obtained ethical approval from the IRB of the Nathan Kline Institute and Montclair State University. Written informed consent or was obtained from adult study participants and written informed consent and assent from minor/child participants and their legal guardian in accordance to local institutional guidelines and with the Declaration of Helsinki.

### 2.2. Study characteristics and MRI acquisition

The study included data from the Nathan Klein Institute (NKI) – Rockland Sample. Anatomical as well as rs-fMRI data of 526 subjects with ages ranging from 6 to 85 years of age were included. FMRI data were acquired using a gradient-echo (GRE) EPI sequence with the following parameters: TR/TE = 1400ms/30 ms; 2 mm isotropic resolution; 64 slices, 112 × 122 matrix size, FA = 65°, total acquisition duration = 10mins. Also, for T1w images, TR/TE = 1900ms/2.52 ms; 1 mm isotropic resolution; 176 slices, 256 × 256 matrix size, FA = 9°, total acquisition duration 4 min and 18 s. The enhanced NKI project was aimed at creating a large-scale (number of participants > 1000) community sample of participants across the lifespan (Nooner et al., 2012; Tobe et al., 2022).

During resting-state fMRI acquisition, participants were awake and instructed to remain still while maintaining visual fixation (eyes open). Pediatric participants were scanned without anesthesia; instead, behavioral preparation and motion-reduction procedures were used to facilitate compliance during MRI acquisition.

### 2.3. Data pre-processing

Data pre-processing and denoising were performed in CONN-toolbox.

Anatomical T1w data were normalized to MNI standard space. We used tissue segmentation to create gray matter (GM), white matter (WM) and cerebro spinal fluid (CSF) masks. Individual brains were further parcellated into 133 cortical gray matter regions of interest (ROIs) based on the Harvard-Oxford atlas. Brain stem, cerebellum, and cerebellar vermis regions were excluded. The remaining 105 cortical brain regions were used for regional complexity analysis (details below).

Functional MRI data pre-processing included motion realignment, coregistration to T1-weighted anatomical images, and normalization to MNI standard space. Additionally, the data were high-pass filtered, and motion parameters with derivatives, as well as cerebrospinal fluid (CSF) and white matter (WM) signal fluctuations, were regressed out using a-CompCorr. Finally, the data were spatially smoothed. Twenty-two subjects with a composite motion score greater than 0.5 mm were excluded from the study. Subjects excluded for excessive head motion were disproportionately younger, particularly children ≤12 years old, who exhibited substantially higher exclusion rates relative to other age groups. This pattern is consistent with prior developmental fMRI literature demonstrating increased in-scanner motion in pediatric populations. The remaining cohort consisted of 504 subjects. For age and sex distribution of the study sample and excluded participants see supplemental material Text S1, Figure S1 and Tables S1 & S2).

The used public dataset showed unequal distribution of age and sex with most subject being adults and lower representation of very young and very old individuals. Furthermore, the female group had better sampling of older individuals as compared to the male group. Unequal sampling across groups may reduce statistical power and affect stability of estimated trajectories, particularly when certain groups are underrepresented.

To further characterize our sample, we performed a data driven exploration of age groups to investigate whether commonly observed life periods emerge from the data itself. Participant age groups were derived using a data-driven hierarchical clustering approach applied to chronological age values from the full cohort. Agglomerative hierarchical clustering was performed in MATLAB (MathWorks, Natick, MA) using average linkage and Euclidean distance metrics. Initially, participants were partitioned into four primary age clusters. Inspection of the resulting clusters demonstrated that the youngest cluster encompassed a broad developmental interval (6–25 years). To improve developmental resolution within this range, a secondary hierarchical clustering analysis was performed exclusively on this subset, specifying two additional subclusters. This resulted in five final empirically derived age groups spanning the lifespan: 6–13 years, 14–25 years, 26–38 years, 39–63 years, and 64–85 years (Table 1).

These age strata were generated empirically from the cohort data rather than imposed using predefined developmental categories. For interpretive purposes, the clusters were considered to broadly correspond to childhood/early adolescence, adolescence/emerging adulthood, early adulthood, middle adulthood, and older/late adulthood, respectively.

### 2.4. Sample entropy (SE)

Sample entropy (Richman and Moorman, 2000) is a variant of Approximate Entropy (ApEn) (Pincus, 1995) that was developed for short and noisy physiological signals. Sample entropy for a given two embedding dimensions can be calculated using

(1) $$SampEn(m,r,N)=-\log\frac{C^{m+1}(r)}{C^{m}(r)},$$

where m (pattern matching length) denotes the length of the block or pattern to be compared, r (pattern matching threshold) is the distance threshold, and N is the length of the time series. The parameters m and r must be chosen, and $C^{m}(r)$ the term in Eq (1) calculates the average conditional probability of recurring data points within the chosen pattern matching threshold (R. X. Smith et al., 2014).

(2) $$C^{m}(r)=\frac{1}{\left(N-m\right)}\sum_{i,j=0}^{N-m+1}\frac{\Theta \left(r-\;\|\;u_{i}^{m}-u_{j}^{m}\;\|\right)}{\left(N-m+1\right)},$$

where $u_{i}^{m}$ and $u_{j}^{m}$ are two embedding dimensions with pattern matching length m and Θ is the Heaviside function.

### 2.5. Multiscale sample entropy (MSE)

Sample entropy evaluates the complexity of a time series on a single scale, whereas MSE evaluates complexity by using multiple coarse-grained time series (Costa et al., 2005). This aids in filtering out the high-frequency fluctuations (R. X. Smith et al., 2014). A new coarse-grained time series is created by averaging consecutive time points of the original time series within non-overlapping windows. For a given time series $\left\{x_{1},\ldots ,x_{N}\right\}$, the averaged time point ($y_{i}^{\tau }$) of the new time series given by

(3) $$y_{i}^{\tau }=\frac{1}{\tau }\sum_{i}^{i+\tau -1}x_{i},$$

where τ is the time scale that determines the number of consecutive time points and the length of the new time series is N∕τ. Accordingly, scale 1 is the original time series, and the sampling frequency (Hz) is 1/TR. Subsequent higher scales will thus have lower frequencies defined as 1/(scale*TR).

MSE was calculated as the area-under-the-curve across SampEn up to τ = 13 scales, with pattern-matching length m = 2 and pattern-matching threshold r = 0.3 (Roediger et al., 2024; Yang et al., 2018). With increasing scale, the number of data points is reduced; therefore, the validity of the SampEn estimates across scales was tested, and robust estimates were demonstrated up to scale 13 (see supplemental material Text S2 and Figure S2).

Voxel-wise resting state fMRI-complexity maps were estimated using an in-house complexity toolbox, LOFT Complexity Toolbox (github.com/kayjann/complexity). Regional complexity for average global gray matter (GM) and 105 cortical areas were calculated for each subject.

### 2.6. Statistical analysis

We evaluated trajectories for global GM complexity as well as regional trajectories of rMRI-complexity and further investigated sex differences in these trajectories. To determine the trajectory of the global GM complexity changes across the lifespan, we applied five models for Bayesian Information Criterion (BIC) based model selection: linear, quadratic, cubic, quartic, and double exponential functions (see supplemental material Text S3, Table S3, and Figure S3). The BIC was calculated using MATLAB to determine the best model fitting for the data. Peak age of complexity maturation in whole, female, and male groups was determined by running stratified bootstrapping analyses for 1000 iterations with 20 age bins for stratification. The BIC model selection was applied in every iteration to determine the best-fit model. Bootstrap iterations that do not successfully model the data using the aforementioned list of polynomials and data with linear distribution from age 6 were ignored during calculations. Confidence intervals (percentile method) and the mean of the peak age of complexity maturation were determined using the peak age distributions obtained from bootstrapping. A similar approach was applied to regional analyses to assess the best-fit model for the data distribution of each ROI by applying the five models: linear, quadratic, cubic, quartic, and double exponential functions, along with BIC. Peak age of complexity maturation was calculated using the fitted models. Rates of increase and decrease were calculated by dividing the datasets into two: pre-peak data and post-peak data. Using linear models, the slope percentages ((Δy∕Δx)×100) before and after the peak were calculated. Finally, K-means clustering was applied to cluster the peak age of complexity maturation and slopes of increase and decrease of complexity over the lifespan to reveal patterns or networks with similar trajectories. All analyses were repeated by separating the cohort into female and male to assess sex-specific trajectories. Three-dimensional (3D) rendered images were created for data visualization of cortical areas using the Quantitative Imaging Toolkit (QIT) software (Cabeen et al., 2018).

To evaluate the potential impact of gray matter (GM) volume on the analysis we calculated the GM volume of each subject using FMRIB Software Library (FSL) and added GM volume as a covariate in a linear model of complexity vs age (see supplemental material Text S4, Figure S4 and Table S4). We also estimated the association of head motion with age as well as potential association between head motion and estimated MSE (supplemental material Figure S5).

### 2.7. Delis-Kaplan executive function scores (D-KEFS)

The NKI study participants underwent neurocognitive testing using Delis-Kaplan Executive Function System test (D-KEFS) (Delis et al., 2012). The d-KEFS is a neurophysiological test which is widely used to test cognition and executive function and consists of 9 stand-alone tests: Color-word Interference Test (CWIT), Design Fluency Test (DFT), Proverb Test (PT), Sorting Test (ST), Tower Test (TT), Trail Making Test (TMT), Verbal Fluency Test (VFT), Word Context Test (WCT), and Twenty Questions Test (TQT). We specifically focused on executive function for this lifespan analysis and did not consider other cognitive domains. After careful consideration of clinical applications of d-KEFS we have chosen 6 primary tests for the analysis: TMT (Number-Letter Switching), VFT (Letter Fluency and Category Switching Total: Number of Correct Switches), CWIT (Inhibition and Inhibition/Switching), and DFT (Switching). TMT aims at assessing set-shifting, attention, processing speed, and visuomotor scanning (Mace et al., 2019; Waggestad et al., 2023). VFT aims at assessing category fluency, set-shifting, and letter fluency (Mace et al., 2019). CWIT measures are effective in assessing working memory, inhibitory control, cognitive flexibility, semantic activation, and response selection (Mace et al., 2019). DFT aims at assessing planning/initiation, cognitive flexibility/divergent thinking, and fluency in generating visual patterns (Mace et al., 2019; Suchy et al., 2010). In TMT, CWIT, and DFT tests, longer completion times generally reflect poorer task performance and may be indicative of underlying cognitive deficits. In contrast, for measures such as VFT total correct, performance is better assessed by the number of correct responses rather than completion time.

After comparing subject IDs with phenotypic data and neuroimaging data, 494 participants had sufficiently complete cognitive test score data. TMT-Number-Letter Switching and DFT-Switching had no missing values, while the remaining four tests had 8 missing values, and those missing values were imputed using spline interpolations. The continuous values in the six tests were standardized to z scores. Statistical analysis between complexity and cognitive test scores in global and regional data was calculated using Spearman’s rank correlation coefficient. Since both complexity and cognitive function are expected to be age-dependent, correcting for age in this analysis could remove the effect; therefore, no age correction was performed. Our rationale for not covarying for age was that age-related variation constitutes a central feature of the biological phenomenon under investigation rather than a nuisance variable. Specifically, both cortical complexity and executive abilities demonstrate nonlinear maturation during development followed by decline in later life, and removing age-associated variance may therefore eliminate meaningful lifespan-related signal.

In supplemental material we present additional sensitivity analyses using an alternative cortical parcellation based on a functionally derived atlas (Schaefer 200 parcel), as compared to the structural segmentation of the Harvard-Oxford atlas. We further present age-adjusted results for this analysis to demonstrate that including age as a covariate reduces the observed lifespan effect (supplemental material Figures S8 & S9).

### 2.8. Analysis of fMRI-complexity and D-KEFS scores within and between data driven age groups

Finally, to corroborate the results across the whole age range, we performed one-way ANOVAs followed by post-hoc tests on complexity and d-KEFS scores separated by age groups that were derived by k-means clustering as described above and listed in Table 1.

## 3. Results

### 3.1. Trajectories for whole-brain gray matter functional complexity

**Fig. 1.**
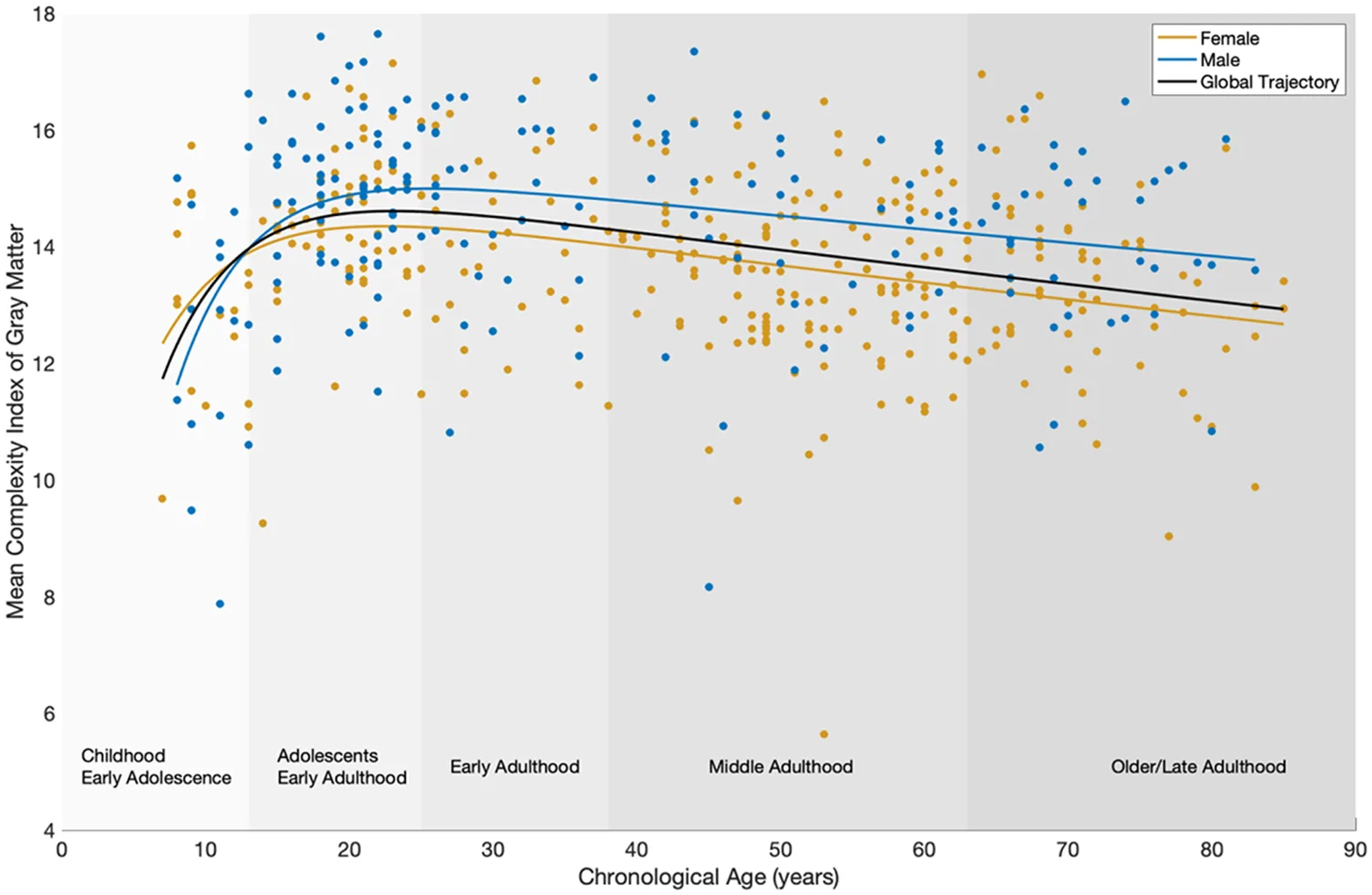
Global trajectory of complexity (Black). Fitted double exponential function for complexity trajectory of female and male given by yellow and blue lines. Mean complexity index of gray matter <7 were excluded from the polynomial fitting.

For the global trajectory of complexity, the lowest BIC was observed for the double exponential function. The peak of the global trajectory of mean MSE occurs at the age of 23.76 years ([21.27, 26.38 years], CI 95%). Before the age of 23, complexity and age have a positive correlation coefficient r = 0.3418 (p = 5.653 × 10^−5^). After the age of 23, the correlation coefficient was r = −0.247 (p = 2.170 × 10^−6^). Regarding sex effect, the complexity peak in females occurs at the age of 23.67 ([20.16, 29.18 years],95% CI) years, whereas for males, the peak age occurs at 26.07 years ([19.95, 33.14 years], CI 95%) (Fig. 1). Generally, after the age of 23, complexity shows a steady decline with age in the young adult, middle-aged, and older adult groups.

There was no significant effect of GM volume on mean MSE, and the recorded p-value was 0.995 (see supplemental material Table S2). However, we found a small but significant association between MSE and head-motion (FD) (r = −0.20 p < 1e-5) as well as between age and head motion (FD) (r = 0.29 p < 1e-10). This relation between residual head motion and age or complexity could introduce bias in the estimated lifespan trajectories. We tested this by calculating the lifespan trajectory for FD-adjusted values (Supplemental Figure S2. We found that raw and adjusted trajectories are very similar, and that peak age of FD-adjusted trajectory is almost identical: 23.33 years (supplemental material Figure S5).

### 3.2. Peak age of complexity

**Fig. 2.**
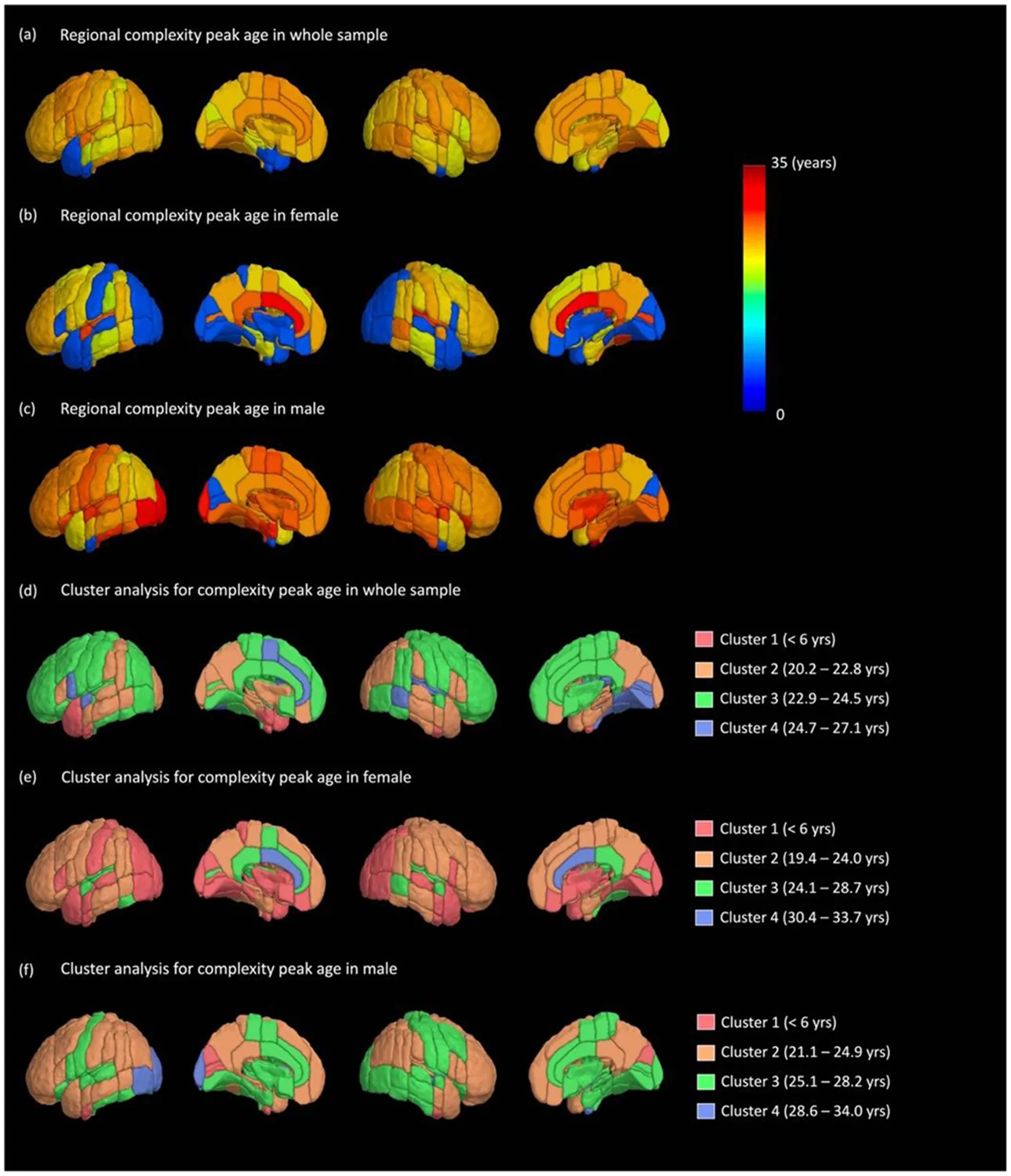
Regional peak age when maximal cortical complexity is measured in global (a), women (b) and men (c). Clusters of peak age of complexity maturation in global (d), women (e) and men (f). Complexity peak age of women in cortical areas varies from 19.4 to 33.7 years (e). In men this range varies from 21.1 to 34 years (f). Cluster 1 represents peak age for ROIs that have a linear decline from age 6.

Qualitative observations indicate that frontal and prefrontal cortical areas reach their peak age later than the parietal cortical areas. In the whole sample, the peak age of complexity in most frontal and parietal regions, representing higher association cortex, occurs after 22 years (Fig. 2a), whereas the occipital and temporal areas show a peak age before 20 years. This pattern is similar and even more pronounced in the female group (Fig. 2b), while the male group shows overall later peak ages (Fig. 2c). Notably, the peak age of complexity among female participants ranges from 19.4 to 33.7 years, whereas for male participants, it varies from 21.1 to 34 years.

To identify distinct cortical areas that undergo similar developmental trajectories and patterns of decline, we performed clustering analysis to group cortical regions based on their fMRI-complexity profiles across the whole cohort, with analyses also separated by sex. We selected four clusters, consistent with previous reports describing distinct spatiotemporal patterns of cortical development (Lynch et al., 2024).

In the whole-sample cluster analysis (Fig. 2d), the left temporal lobe shows a complexity peak age below 6 years. In contrast, most regions in the frontal lobe and anterior cingulate gyrus exhibit substantially later peak ages of complexity.

In the female group, the complexity peak in regions of the occipital pole and temporal pole falls into the below-6 years age group, as the observed data trend was linear from age 6 to 85 years (Fig. 2e). In males, however left hemisphere occipital region reaches peak age of complexity later than other regions (28.6 – 34 years) (Fig. 2f). Overall, both male and female groups showed a later peak of complexity in the frontal cortex and anterior cingulate gyrus.

### 3.3. Rate of increase and decrease of complexity over the lifespan

**Fig. 3.**
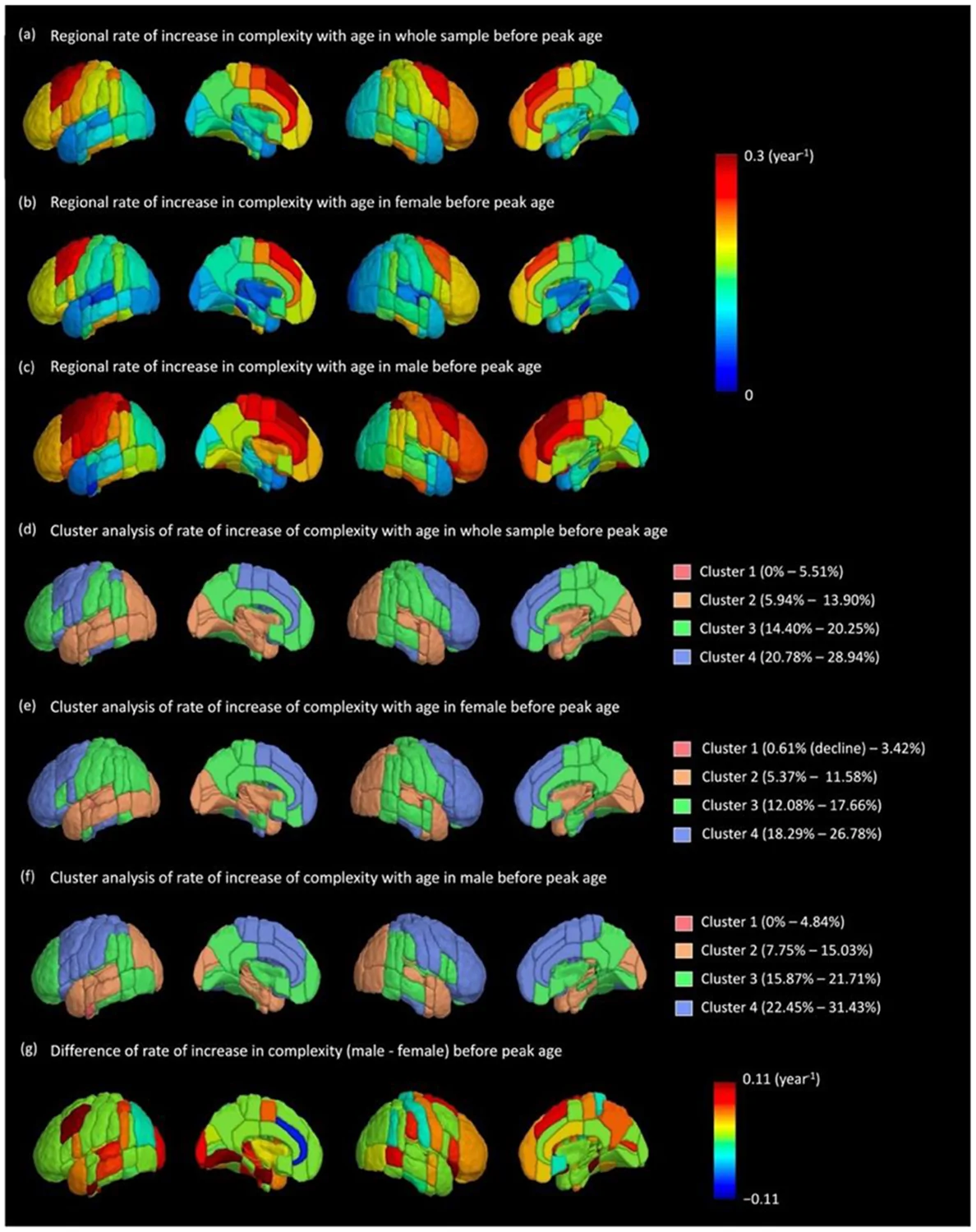
Change of cortical complexity before peak age. (a) Increase of cortical complexity with age (global). (b) Increase of cortical complexity with age in women. (c) Increase of cortical complexity with age in men. Clusters of rates of increase of complexity with age in global (d), women (e) and men (f). (g) Difference of rate of increase in complexity between male and female. Maximum and minimum values of slope percentage for each cluster are shown in the legend.

Before the global peak at age 23, the rate of increase in complexity with age in frontal and parietal lobes is higher than the rest of the brain areas (Fig. 3a). This observation prevails in male and female groups (Fig. 3b & 3c).

The cluster analysis clearly separates occipitotemporal (Cluster 2) from parietal (Cluster 3) and from frontal cortex (Cluster 4). These clusters define different rates of increase in adolescents to early adulthood, with Cluster 4 showing a more rapid increase than Cluster 3 and Cluster 2, respectively.

The frontal lobe of the right hemisphere has the highest rate of increase in complexity compared to other regions in the whole sample, female, and male groups (Fig. 3d, 3e, 3f). In the male group parietal lobe of the left hemisphere has the highest rate of increase in complexity. In the female group, 104 ROIs showed positive slopes, except the transverse temporal gyrus, which had a negative slope (Cluster 1, Fig. 3e). Overall, the male group shows a higher rate of increase in complexity compared to the female group in all clusters (Fig. 3c). Fig. 3g illustrates the magnitude of the difference in slope between the male and female groups for each ROI (male - female). Except for the temporal pole and the middle temporal gyrus, the male group has a higher rate of increase in complexity compared to the female group (Fig. 3g). However, these sex specific effects are not statistically significant after correction for multiple comparison.

**Fig. 4.**
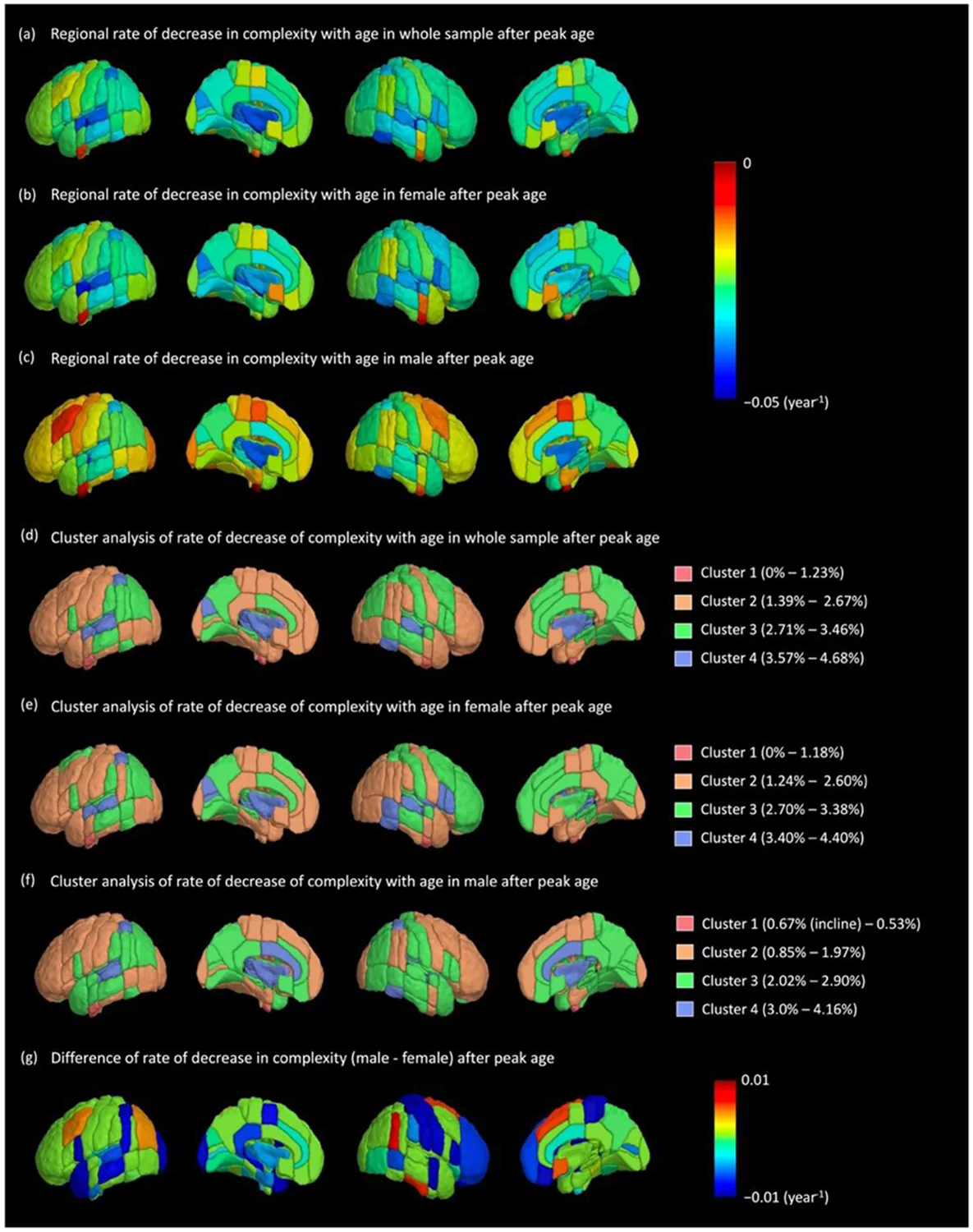
Change of cortical complexity after peak age. (a) Decrease of cortical complexity with age (global). (b) Decrease of cortical complexity with age in women. (c) Decrease of cortical complexity with age in men. Clusters of rates of decrease of complexity with age in global (d), women (e) and men (f). (g) Difference of rate of decrease in complexity between male and female. Maximum and minimum values of slope percentage for each cluster are shown in the legend.

After reaching a peak in complexity at age 23, a moderate rate of decrease between 0 and 0.05 per year is shown in most of the brain regions in both the whole sample and the female group (Fig. 4a and 4b). However, in the male group, frontal and anterior parietal lobes show a slower rate of decrease compared to the rest of the regions (Fig. 4c).

In cluster analysis prefrontal cortex and central gyri of the left hemisphere showed a rate of decrease of complexity between ~1% - 2.67% and this result is consistent in the whole sample, female, and male groups (Fig. 4d, 4e, & 4f). The female group showed a faster rate of decline in the frontal lobe of the right hemisphere compared to the left hemisphere male group (Fig. 4e). In the male group, both right and left frontal lobes exhibit a slower decline compared to other brain areas (Fig. 4f). The fastest rate of decline in the female group is mainly in the insular cortex, and in the male group anterior cingulate gyrus and the insular cortex both have the highest rate of decline. Also, 104 ROIs in the male group showed a negative slope, except for the temporal fusiform cortex, which had a positive slope (Cluster 1, Fig. 4f). Overall, the female group showed a faster rate of decline in all clusters compared to the male group. The magnitude of the difference in the rate of decrease between the male and female groups is illustrated in Fig. 4g Eighty-two percent of brain areas showed a higher rate of decline in complexity in females compared to males (Fig. 4g).

In general, we observed a counterpose of results between males and females when comparing results before peak age and after peak age. These sex-specific observations on the rate of decrease of complexity are not statistically significant.

### 3.4. Cognitive scores and complexity

We observed a strongly significant negative correlation with global fMRI-complexity in TMT-Number-Letter Switching, VFT-Letter Fluency, CWIT-Inhibition, and CWIT-Inhibition/Switching (Table 2). TMT-Number-Letter Switching, CWIT-Inhibition, and CWIT-Inhibition/Switching have a significant correlation with complexity. Other d-KEFS tests did not show significant correlation with complexity. The goal of the correlation analysis was to observe the existence of a monotonic relationship between fMRI complexity and d-KEFS scores. Spearman’s rank correlation coefficient is suitable when data are heavy-tailed, include outliers (de Winter et al., 2016), or to observe a monotonic relationship in continuous data. However, Pearson correlation of fMRI-complexity and d-KEFS was also calculated, and a similar outcome of significance was observed (see supplemental material Figure S6 and Table S5).

In supplemental material (Text S4 and Figure S&) we further present the correlation between the separate tests as well as a factor analysis showing that there is sufficient variance between test to justify separate association analyses with cortical complexity.

**Fig. 5.**
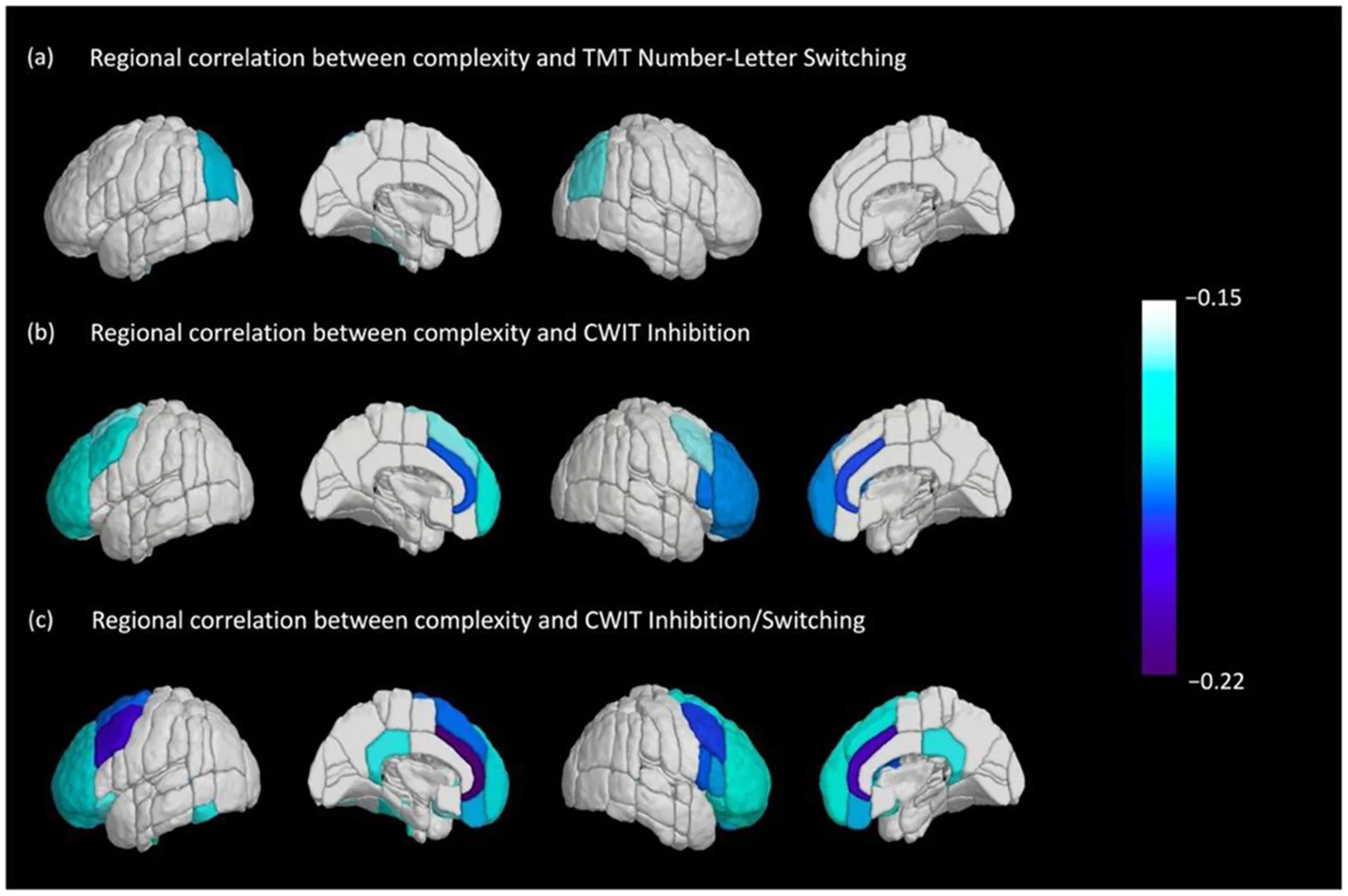
Spearman’s rank correlation coefficients between regional complexity and d-KEFS scores, significant after correction for multiple comparisons (adjusted *p* value 0.00047). (a) Correlation between complexity and TMT Number-Letter Switching. (b) Correlation between complexity and CWIT Inhibition. (c) Correlation between complexity and CWIT Inhibition/Switching.

In regional analysis of fMRI-complexity and d-KEFS scores, TMT Number-Letter Switching showed significant negative correlation in parietal and medial temporal lobe (Fig. 5a), whereas both CWIT Inhibition and CWIT Inhibition/Switching showed significant negative correlation with fMRI-complexity in lateral and medial frontal cortex (Fig. 5b and 5c). Among CWIT inhibition and CWIT inhibition/Switching, the later showed overall stronger negative correlation than CWIT inhibition. Interestingly, CWIT Inhibition/Switching also showed correlation in posterior cingulate cortex and temporal lobe.

Supplemental Figures S8 and S9 depict a replication of these findings using a functionally derived parcellation (Schaefer atlas) as well as results adjusted for age and age^2^. Notably, adjustment for age reduced the observed effect but findings for CWIT test remain significant, thus demonstrating that there is an association between complexity and executive function beyond a mere age effect. However, the main hypothesis of this study was to test whether there is an joint maturational trajectory of complexity and executive function across age stages.

### 3.5. Test of complexity and cognitive score for the data driven age groups

There is an inverted trajectory in d-KEFS test scores across age groups compared to the trajectory of complexity across age groups. The complexity peak maturation in global, female, and male groups occurs in the adolescent and young adult age groups, whereas the lowest time recorded in completing tasks in three d-KEFS tests is also associated with the adolescent and young adult age groups.

## 4. Discussion

**Fig. 6.**
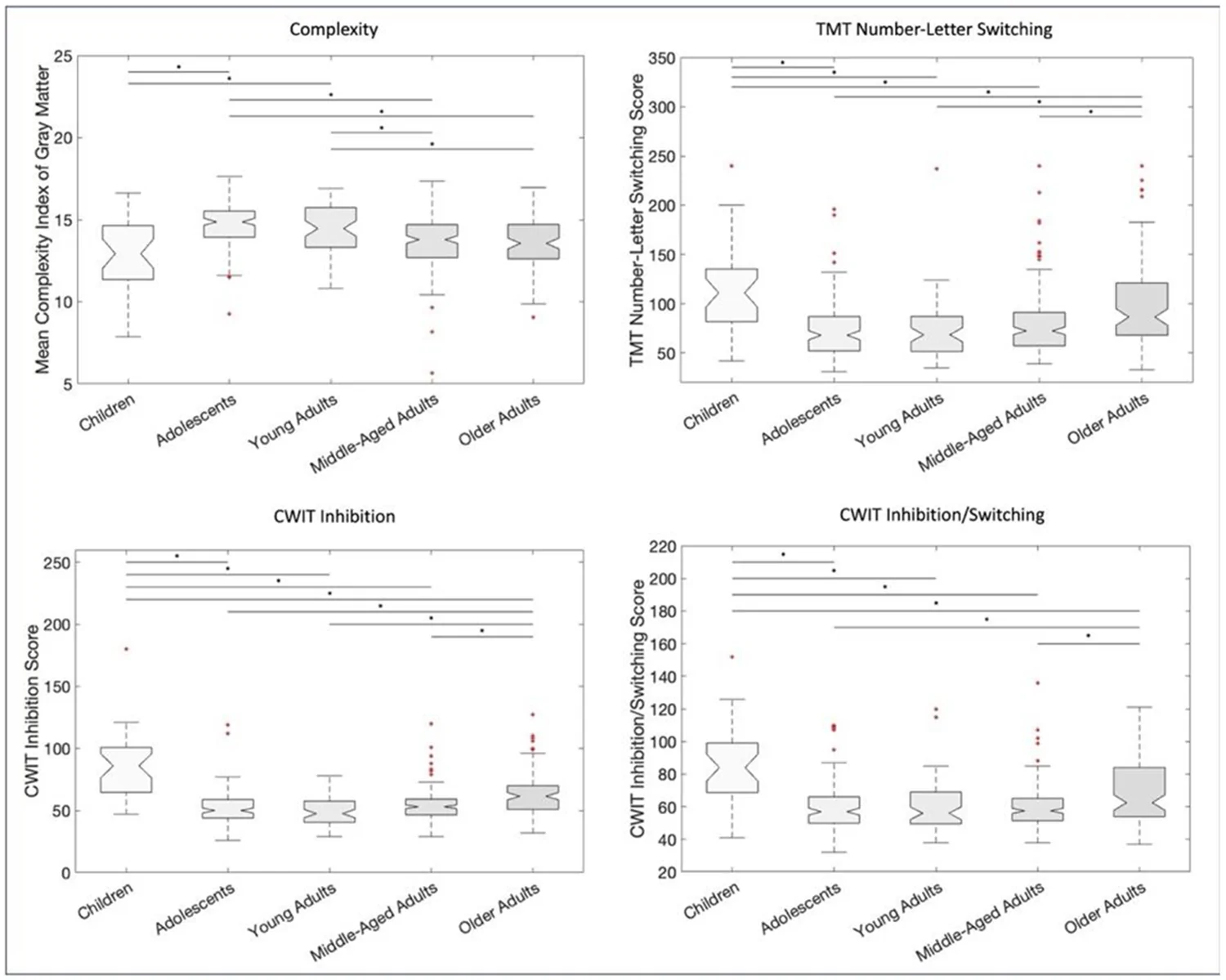
Multiple comparison tests among age groups (a) Complexity vs age (b) TMT Number-Letter Switching score vs age (c) CWIT Inhibition score vs age (d) CWIT Inhibition/Switching score vs age. (* statistically significant age group pairs (*p* value 0.05)).

In this study, we present a comprehensive characterization of cortical trajectories of MRI-complexity across the human lifespan using MSE. Lifespan age groups were identified using a data-driven approach, yielding bins that closely align with standard developmental classifications. Notably, most analyses in this study were performed using age as a continuous variable, except for the results presented in Fig. 6.

Our results show that fMRI-complexity increases rapidly throughout childhood and adolescence, reaches a peak in early adulthood, and then declines gradually into later life (Fig. 1). This overall trajectory is consistent across female and male participants, although males exhibit a maturational delay, reaching peak complexity at older ages than females. Regional analyses revealed a general posterior-to-anterior pattern of maturation, with frontal areas exhibiting peak complexity at a later stage (Fig. 2). This pattern is largely similar across sexes, although some regional differences are evident. As shown in Fig. 2, several brain regions reach peak complexity at age 6 or younger. Since our sample begins at age 6, these estimates reflect the earliest measurable age in the cohort. Previous research supports the notion that substantial maturation occurs before age 6. For example, cortical structure, including gray-matter volume and surface area, develops rapidly in the first years of life, with individual differences largely established by age 1 (Gilmore et al., 2020). Region-specific changes in cortical thickness, surface area, and gyrification have also been observed in children between 1 and 6 years, with some posterior regions showing earlier maturation (Remer et al., 2017).

After the peak, complexity declines at different rates across cortical regions: parietal and temporal areas show earlier and steeper decreases, followed by pre(frontal) regions. Although females appeared to show a somewhat faster post-peak decline (Fig. 4), sex-specific increases or decreases were not statistically significant.

Finally, we also demonstrate that functional complexity is associated with cognitive performance, globally (Table 2), and more specifically, executive function in frontal and parietal cortices (Fig. 5).

### 4.1. Spatiotemporal cortical trajectories of functional complexity

Our findings based on fMRI-complexity align closely with prevailing hypotheses of cortical development, maturation, and later-life decline. The observed trajectories support earlier histological and neuroimaging evidence demonstrating spatially heterogeneous patterns of cortical maturation that reflect cytoarchitectonically determined regional developmental processes. Our results are consistent with the well-established posterior-to-anterior hierarchical gradient of cortical maturation, also referred to as the sensorimotor-association (S-A) axis, which has been documented across electrophysiology, neuroimaging, and histological studies (Sydnor et al., 2021). This axis describes the sensorimotor-association cortical axis (S-A axis) of cortical function, transition from lower-order sensorimotor to highest-order associative functions. While primary sensory and motor function develop early, the association cortex, which supports executive function of the brain, matures well into the second decade of life (Sydnor et al., 2021).

These developmental patterns align with known microstructural processes. The dendritic structure of cortical areas is known to undergo significant changes during early maturation, including increases in dendritic density and synapse number. Furthermore, during neurodevelopment, pruning of dendritic trees is a critical process associated with specialization and efficiency of brain circuits. Alterations to synaptic and dendritic processes are also central to the pathologies of Alzheimer’s disease (AD). Therefore, the microstructure architecture critically determines the electrical communications of neurons and shapes how synaptic signals integrate and support neuronal function. Recent imaging studies further support this framework. For example, Lynch et al. (2024) used diffusion MRI to characterize spatiotemporal patterns of microstructural maturation in children and adolescents, reporting terminal maturation of neurite density index (NDI) in late adolescence across primary sensory (20.96 years), occipitoparietal (18.21 years), frontotemporal (23.95 years), and limbic (19.36 years) regions (Lynch et al., 2024). Complementing these neurobiological findings, behavioral evidence from Gauvrit et al. (2017), who examined human cognitive complexity using random-item generation tasks, demonstrated a similar developmental peak around age 25 (Gauvrit et al., 2017).

Only a few studies have attempted to characterize lifespan changes in functional complexity using alternative mathematical descriptions of complexity. For example, Dong et al. applied Hurst Exponents to a subsample of the NKI cohort (N = 116; ages 19–85 years) and reported age-related decreases in complexity in frontal and parietal regions, alongside increases in other areas, including limbic and occipital areas (Dong et al., 2018). Similarly, Sokunbi et al. (2015) examined Fuzzy Approximate Entropy (fApEn) and SampEn in 86 healthy adults aged 19–85 years and found linear declines in both metrics within frontal and parietal cortices (Sokunbi et al., 2015). Notably, neither study included participants from childhood or adolescence, and both used linear modeling, which limited their ability to capture maturational processes or estimate peak developmental ages.

A more recent study by Niu et al. (2022) examined Permutation Entropy (PermEn) in a broader cohort spanning ages 8–85 years (N = 319) and identified an inverted U-shaped global trajectory peaking at approximately age 40, with network-specific peaks ranging from 25 to 52 years (Niu et al., 2022). Compared to these findings, our results more closely align with established literature on cortical morphology and cognitive development, which consistently show peak maturation in early adulthood.

It is important to note, however, that while (Multiscale) SampEn is among the most widely used measures for characterizing neurodevelopmental conditions (Sokunbi et al., 2013; Zhang et al., 2024) as well as aging and neurodegeneration (Jann et al., 2023, 2024; Niu et al., 2022; R. X. Smith et al., 2018; Z. Wang, 2020; Zhang et al., 2025), many mathematical formulations of complexity exist, and their reliability and sensitivity for fMRI analysis require further systematic evaluation. For instance, Guan, Wan, et al. (2023) compared several entropy measures using both simulated and neuroimaging data. In simulated time series from three statistical distributions, most entropy metrics produced stable curves, whereas SampEn showed instability for Normal and Weibull distributions. When applied to rs-fMRI data, Approximate Entropy, Dispersion Entropy, and Permutation Entropy failed to differentiate healthy individuals from participants with schizophrenia, bipolar disorder, or ADHD. This highlights that not all entropy metrics are equally sensitive to detecting meaningful differences in brain function (Guan, Wan, et al., 2023).

Overall, our study is the first to characterize lifespan trajectories of functional complexity using MSE, thereby expanding the repertoire of tools for studying cortical development and later-life decline. MSE provides a measure of functional complexity that complements established structural markers, such as cortical thickness, volume, microstructure, and myelination.

Finally, it is worth noting that MSE-based findings align with a broader literature on BOLD signal variability. Measures such as Root Mean Square of Successive Differences (RMSSD) and fractional Amplitude of Low-Frequency Fluctuations (fALFF), which capture moment-to-moment fluctuations and frequency characteristics of the BOLD signal, have been linked to cognitive flexibility, neural efficiency, and developmental changes. Studies by Garret et al. and Goodman and colleagues have shown that higher BOLD variability often corresponds to more adaptive neural processing in adolescence and early adulthood, with reductions observed later in life (Garret et al., 2011; Goodman et al., 2024). These converging observations suggest that MSE may reflect a general property of intrinsic neural dynamics that integrates temporal variability and spectral organization across cortical development.

### 4.2. Sex differences in trajectories of functional complexity

Based on the global trajectories, males exhibited a later peak in fMRI-complexity compared to females, a pattern that was also observed in the regional analyses. However, these sex differences were not statistically significant. In the regional analyses separated into male and female participants, the male group showed later peak ages in widespread cortical areas. The cluster analysis highlighted this further: in females, the motor–temporal cluster peaked before 6 years of age, whereas in males, it occurred between 25 and 28 years; the frontal–parietal cluster peaked at 19–24 years in females and 21–24.9 years in males.

Despite these differences in timing, the overall spatial pattern of regions with higher or lower rates of complexity increase was similar across the whole cohort and between sexes, with the steepest increases in frontal areas and lower rates in occipital and temporal regions. Although males generally showed slightly higher rates of increase, sex differences in both the rate of complexity increase and decline were not statistically significant. These non-significant findings might be due to imbalanced sampling of sexes in early and late life which lowers statistical power. Larger samples with equal sampling across age and sex will be needed in the future.

### 4.3. Association of functional complexity with cognition

To examine the relationship between fMRI-complexity and cognitive function, we correlated global and regional complexity with selected d-KEFS test scores. Global complexity was inversely associated with CWIT and TMT performance, both of which assess executive functions including processing speed, cognitive flexibility, and inhibition. Longer completion times on these tests reflect poorer performance, consistent with our hypothesis that higher complexity supports more efficient cognitive processing during maturation, and that age-related declines in complexity correspond to decreases in executive function among healthy subjects.

Consistent with this expectation, prior studies have linked MSE to higher-order cognition (Omidvarnia et al., 2021). Other studies by Wang have examined the relationship between brain entropy and cognition and also found an association in the frontoparietal executive control network (Del Mauro and Wang, 2024; Z. Wang, 2021). The regional analysis for TMT and CWIT revealed that TMT is primarily associated with the parietal lobe and medial temporal lobe, whereas CWIT shows effects in the lateral and medial frontal cortex, and CWIT also includes the posterior cingulate cortex and inferior temporal areas. These frontal, parietal, and temporal regions are central to executive function, and alterations in these brain areas are linked to behavioral and cognitive deficits in neurodevelopmental and neurodegenerative disorders. We replicated these findings using an alternative, functionally derived parcellation to corroborate that the association between executive function and complexity is robust to cortical parcellations (Supplemental Figure S8). Additionally, correcting for age, reduced the observed effect but did not completely remove it (Supplemental Figure S9). This indicates that the association between complexity and executive function persists beyond the maturation across different life stages. Collectively, these findings support the notion that fMRI complexity could be a novel and promising approach to detect and characterize abnormal trajectories, and it holds potential as an early marker of subsequent cognitive decline (Zhang et al., 2025).

### 4.4. Limitations

Several limitations should be considered when interpreting these findings. First, the data are cross-sectional, and longitudinal studies would be preferable for more accurately characterizing lifespan trajectories. While collecting data spanning several decades of life is hardly feasible, even a few years of longitudinal data per participant could improve the estimation of model trajectories. Second, our sample had a skewed age distribution in the male cohort: the middle-aged and older adult groups included fewer male participants compared to females. A similar imbalance is evident in the complexity vs d-KEFS analysis, with 314 female participants and 180 male participants. These unequal sample sizes may have influenced the study results. While estimation of peak-age likely is robust thanks to high representation within that age range, the non-significant sex effects are probably explained by the reduced statistical power due to imbalanced sampling. Furthermore, limitations inherent to the d-KEFS tests remain, such as uncertainties related to time-dependent measures of executive function tasks (Delis et al., 2004).

### 4.5. Conclusions

Our results indicate that early maturation and gradual decline of brain activity can be captured using fMRI complexity. Moreover, fMRI complexity demonstrated both global and regional associations with executive function. These findings suggest that fMRI complexity could serve as a novel and promising tool to detect and characterize atypical neurodevelopment, as well as a potential early marker of cognitive decline in the progression of Alzheimer’s Disease (Grieder et al., 2018; Zhang et al., 2025).

## Supplementary Material

1

Supplementary material associated with this article can be found, in the online version, at doi:10.1016/j.neuroimage.2026.122085.

## Figures and Tables

**Table 1:** Age groups based on k-means clustering of participant age. Groups align mostly with different life stages across the lifespan.

Group	Age (years)	Number of Female/ Male Participants	Total Number of Participants
Childhood/Early Adolescence	6 - 13	17/17	34
Adolescence/Emerging Adulthood	14 - 25	66/64	130
Early Adulthood	26 - 38	32/28	60
Middle Adulthood	39 - 63	141/39	180
Older/Late Adulthood	64 - 85	65/35	100

**Table 2:** Spearman’s rank correlation between complexity and d-KEFS tests. (*significant after correction for multiple comparisons (adjusted *p* value 0.0083)).

	TMT Number-Letter Switching	VFT Letter Fluency	VFT Category Switching Total	CWIT Inhibition	CWIT Inhibition/ Switching	DFT Switching
Correlation	−0.1526	−0.0078	0.0223	−0.1229	−0.1665	0.0239
***p*** value	0.0007*	0.8619	0.6204	0.0073*	0.0002*	0.5963

## Data Availability

The data used in this project are managed by the Nathan Kline Institute and are available upon request. The in-house complexity toolbox can be accessed via Github, github.com/kayjann/complexity.
