# Supplementary material for: Lifespan trajectories of the brain’s functional complexity characterized by multiscale sample entropy
Source: Neuroimage. Author manuscript; Available in PMC 2026 Aug 19. (PMC13489196; doi:10.1016/j.neuroimage.2026.122085)
Supplement: Supplementary file 1 [file NIHMS2200353-supplement-1.docx]

**Supplemental Material**

**Text S1**: Age distribution of the sample

Initially 526 subjects with both resting state fMRI and T1 images were chosen randomly from the NKI sample. Twenty-two subjects with a composite motion score higher than 0.5 mm were removed from the analysis. The remaining 504 subjects included in the study were 321 female and 183 male participants. Figure S1 displays the age distribution for the whole study cohort and female and male groups, respectively.

(a)

(b)

(c)

**Figure S1**: Age distribution of the sample. (a) Age distribution of the whole sample. A Gaussian distribution is fitted to the data ($\mu=42.3135$, $\sigma=21.086$). (b) Age distribution of the female group with a fitted Gaussian distribution ($\mu=42.3135$, $\sigma=21.086$). (c) Age distribution of the male group with a fitted Weibull distribution ($\alpha=42.1254$, $\beta= 1.8322$).

**Table S1:** Comparison of excluded participant age vs thole sample.

| **Group** | **N** | **Mean Age** | **Median Age** | **SD** | **Range** |
| --- | --- | --- | --- | --- | --- |
| Excluded subjects | 22 | 35.6 | 39.5 | 23.2 | 6–77 |
| Whole sample | 526 | 42.0 | 44.0 | 21.2 | 6–85 |

**Table S2:** Comparison of proportion of excluded participants in various age ranges compared to the whole sample. Proportionately more children were excluded, which agrees with the common observation that children tend to move more in the scanner than adults.

| **Age Group** | **Excluded (%)** | **Whole Sample (%)** |
| --- | --- | --- |
| Children (≤12) | 31.8% | 6.3% |
| Adolescents (13–18) | 4.5% | 10.1% |
| Young adults (19–35) | 9.1% | 26.0% |
| Middle adults (36–50) | 27.3% | 19.2% |
| Older adults (51–65) | 18.2% | 20.7% |
| Seniors (66+) | 9.1% | 17.7% |

**Text S2**: Change in GM and CSF complexity across different timescales.

We expect CSF to exhibit primarily random noise signal fluctuations, while GM demonstrates more complex behavior. Therefore, Sample Entropy (SampEn) should be significantly higher in GM than in CSF. Using a paired-sample two-sided t-test at each scale, we show statistically significant differences across all scales included in the MSE estimation. Furthermore, GM complexity exhibits scale-invariant behavior, which is typical of a complex system.

**Figure S2**: Change in GM and CSF across different timescales of multiscale sample entropy (MSE). * indicates statistical significance (paired sample, two sided, t-test) between GM and CSF (*p < 0.05*).

**Text S3**: Bayesian Information Criterion (BIC) for model fitting

For the ROI analysis of 105 regions, we applied five functions for each ROI to evaluate the lowest BIC. For the total cohort, we observed 6 regions with the lowest BIC values for linear fit, 31 regions with quartic fit, and 68 regions with double exponential fit. We observed no region with quadratic and cubic polynomial fits. For female subjects, we observed 46 regions with linear fit, 3 regions with quadratic fit, 9 regions with quartic fit, and 47 regions with double exponential fit. For male subjects, there were 7 regions with linear fit, two regions with cubic and quartic fits, and 96 regions with double exponential fit (Table S3). Among the three categories of the analysis (global, female, and male), most ROIs exhibited a double exponential trajectory depicting an initial increase in complexity followed by a decrease after a peak. Contrary, regions that have a linear association with age show primarily a decrease in complexity after the age of 6 years. In Figure S3, we display (a) exemplary trajectories for different models and (b), (c), and (d) the best model for each cortical area for each of the grouped analyses.

**Table S3**: Number of ROIs for each polynomial fit

| Polynomial | Number of ROIs  (C index - Global) | Number of ROIs  (C index - Female) | Number of ROIs  (C index - Male) |
| --- | --- | --- | --- |
| Linear | 6 | 46 | 7 |
| Quadratic | 0 | 3 | 0 |
| Cubic | 0 | 0 | 1 |
| Quartic | 31 | 9 | 1 |
| Double Exponential (Exp (2)) | 68 | 47 | 96 |

**Figure S3**: Polynomial fitting with Bayesian Information Criterion (BIC). (a) Linear, quartic, and double exponential fit for temporal pole (left), frontal pole (right), and superior frontal gyrus (right) for global complexity index. (b) Polynomial fitting in global complexity index vs age. (c) Polynomial fitting in complexity index (female) vs age. (d) Polynomial fitting in complexity index (male) vs age.

**Text S4**: Effect of gray matter volume on complexity analysis

Gray matter volume of each subject was calculated using the FMRIB Software Library (FSL). By applying FSL command “fast” on T1 images, images were segmented into tissue compartments, and the “fslstats” command was used for tissue volume quantification. This provides partial volume estimates for the volume quantification. Gray matter volume decreases with age. This can influence the results obtained for the mean gray matter complexity vs age distribution. Therefore, to evaluate the influence of gray matter volume in our analysis, a generalized linear regression model (MATLAB ‘fitglm’) was applied among complexity, age, and mean gray matter volume as a covariate (Table S4).

**Figure S4**: Gray matter volume vs chronological age in years (female in yellow and male in blue).

$$Mean gray matter complexity \sim1+ Age+Mean\_graymatter\_volume$$

**Table S4**: Results of the generalized linear regression model

|  | Estimate | SE | t-statistic | P value |
| --- | --- | --- | --- | --- |
| Age | $-0.015478$ | $0.005586$ | $-2.7708$ | $0.0058$ |
| Mean Gray matter Volume | $-6.0477\times{10}^{-9}$ | $1.1824 \times{10}^{-6}$ | $-0.0051$ | $0.9959$ |

*Standard Error (SE)

**Figure S5**: Comparison of trajectories estimated based on raw data or after adjustment for head-motion (FD).

**Figure S6**: D-KEFS test scores vs Age ((a) – (f)). Bayesian Information Criterion (BIC) was used to choose the best polynomial. (a) TMT Number-Letter Switching vs age fitted with a quadratic polynomial. (b) VFT Letter fluency vs age, (c) VFT Category Switching scores vs age, (e) CWIT Inhibition vs age and (f) CWIT Inhibition/Switching vs age fitted with cubic polynomials. (d) DFT Switching vs age. Polynomial fitting is not applied on DFT Switching vs age distribution. Mean gray matter complexity vs D-KEFS test scores ((g) – (l)). Spearman’s rank correlation coefficients are displayed on each figure panel.

**Table S5**: Pearson correlation coefficient between complexity and D-KEFS tests. (*significant after correction for multiple comparisons (adjusted $p$ value 0.0083))

| D-KEFS Test | Pearson Correlation | P value |
| --- | --- | --- |
| TMT -Number-Letter Switching | -0.1262 | 0.0050* |
| VFT - Letter Fluency | -0.0011 | 0.9811 |
| VFT - Category Switching Total | 0.0136 | 0.7629 |
| CWIT - Inhibition | -0.1200 | 0.0076* |
| CWIT - Inhibition/Switching | -0.1787 | 0.0000648* |
| DFT - Switching | 0.0301 | 0.5051 |

**Text S4** Analysis of shared variance or independent cognitive constructs of DKEFS scores.

Cross correlation analysis showed that some tests shared substantial common variance (eg CWT with TMT), while others showed moderate correlations (eg VFT and DTF). There was moderate inverse relation between CWT/TMT and VFT/DFT. Overall, we selected test categories that probe for broader executive control but did not test for other cognitive domains.

These findings of moderately shared variance between cognitive tests in our cohort is in general agreement with the findings reported in the D-KEFS technical manual

[Delis, D. C., Kaplan, E., & Kramer, J. H. (2001). Delis–Kaplan Executive Function System (D-KEFS): Technical manual. The Psychological Corporation]

**Figure S7**: Cross-Correlation between scores of the 6 D-KEFS tests.

To check the shared variance specifically we performed a PCA and found that the first component explains 49.9 percent of the shared variance across the 6 tests. The individual tests loaded TMT 0.43, VFT1 -0.38, VFT2 -0.38. DFT -0.29, CWT1 0.47, CWT2 0.46. These loadings agree with the cross correlation above. It also shows that there is sufficient un-shared variance to justify separate analyses for each test.

**Figure S8**: Regional Correlation between fMRI-complexity and D-KEFS scores for the functionally derived Schaefer atlas.

Findings corroborate the findings reported in the manuscript for a structurally derived Harvard-Oxford atlas.

**Figure S9**: Regional Correlation between fMRI-complexity and D-KEFS scores including adjustment for age and age^2^ for the functionally derived Schaefer atlas.
Findings show that including age reduces the effect, which was hypothesized since both, complexity and executive function, mature with age and thus have age-dependence. However, these findings also demonstrate that there is a relation between complexity and executive function beyond the lifespan maturational effect.
